# Proteomic Characterization of Major Fish Allergy Responsive Protein Parvalbumins in Hilsa (*Tenualosa ilisha*): A Commercially Important Fish in Southeast Asia

**DOI:** 10.1021/acs.jafc.5c04335

**Published:** 2025-11-17

**Authors:** Nazma Shaheen, Zongkai Peng, Amit Singh, Asfia Wahab, Maria Gasset, Md. Hafizul Islam, Oumma Halima, Aleena F. Ali, Salmaan M. Shah, Malek Salkini, Saaim Saleemi, Abdullah F. Mallah, Ayan Khan, Sohaib Mesiya, Morshed Khandaker, Aayan Zarif, Akbar Ali, Amir Samour, John W. Peters, Zhibo Yang, Nagib Ahsan

**Affiliations:** † Institute of Nutrition and Food Science, 95324 University of Dhaka, Dhaka 1000, Bangladesh; ‡ Department of Chemistry and Biochemistry, 6187 The University of Oklahoma, Norman, Oklahoma 73019, United States; § Department of Biology, 98462 University of York, York YO10 5DD, U.K.; ∥ Institute of Physical-Chemistry Blas Cabrera,Spanish National Research Council, Madrid 28006, Spain; ⊥ Dodge Family College of Arts and Sciences, University of Oklahoma, Norman, Oklahoma 73019, United States; # Department of Biology, 3140 University of Central Oklahoma, Edmond, Oklahoma 73034, United States; ∇ Nanobiology Laboratory, School of Engineering, 3140 University of Central Oklahoma, Edmond, Oklahoma 73034, United States; ○ Mercy School Institute, Edmond, Oklahoma 73013, United States; ◆ Department of Biochemistry and Physiology, 194751 The University of Oklahoma Health Sciences Center, Oklahoma City, Oklahoma 731034, United States; ¶ Mass Spectrometry, Proteomics and Metabolomics Core Facility, Stephenson Life Sciences Research Center, 6187 The University of Oklahoma, Norman, Oklahoma 73019, United States

**Keywords:** allergens, developmental stage, Clupeidae, food safety, targeted proteomics

## Abstract

Parvalbumin-beta (PRVB) is the major allergen in bony fish; however, its characteristics in Hilsa, an economically important anadromous fish in Southeast Asia, remain unclear. This study characterized Hilsa PRVs using proteomic approaches across two major riverine habitats and developmental stages. Three PRV isoforms were identified in Hilsa muscle through unique peptide sets. Label-free quantitative proteomics, supported by ELISA, revealed higher PRVB levels in juvenile Hilsa compared with adults, independent of habitat. Comparative analysis showed that Hilsa contained significantly lower PRVB than other freshwater species. Additionally, multiple reaction monitoring (MRM)-based targeted proteomics demonstrated PRV’s potential as a marker for allergen quantitation and for authenticating Hilsa in mixed fish samples. These findings provide novel insights into Hilsa PRVs, offering a foundation for future studies of fish quality, allergen assessment, and food safety.

## Graphical abstract

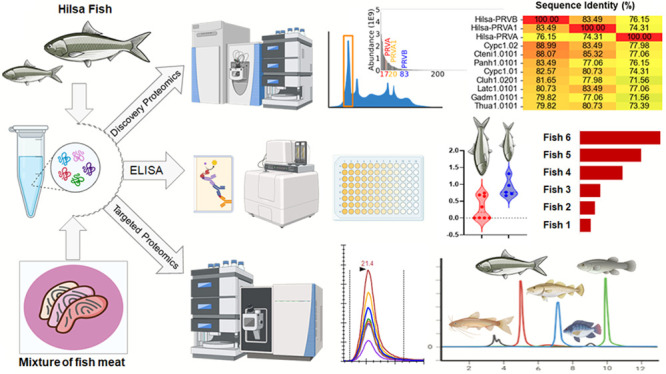

## 1 Introduction

Fish allergies, particularly those triggered by the protein parvalbumin, are a significant global health concern.^1^ Parvalbumin (PRV), particularly parvalbumin-beta (PRVB), is a calcium-binding protein predominantly found in the muscle tissue of bony fish and is responsible for nearly 90–95% of fish-induced allergic reactions.^1–3^ Its unique stability with high resistance to heat, digestion, and denaturation makes it a potent allergen, capable of triggering immune responses even after fish is cooked.^4^ Cross-reactivity among fish species is a significant concern for individuals with fish allergies, as PRVB exhibits a high degree of structural similarity across different species.^3,5–8^ This cross-reactivity means that over 90% of fish-allergic individuals will likely experience allergic reactions to multiple fish species.^5,8^ In contrast, cartilaginous fish, such as sharks and rays, primarily express parvalbumin-alpha (PRVA), a form that is much less allergenic.^1,9^

Hilsa (*Tenualosa ilisha*), the national fish of Bangladesh, is often referred to as the “king of taste” due to its rich, buttery flavor and high nutritional value, particularly its protein content and omega-3 and omega-6 fatty acids.^10^ It plays a central role in the culinary traditions of Bangladesh, where it is both a dietary staple and an integral part of national celebrations. About 90% of the world’s Hilsa production comes from Bangladesh, making it the largest Hilsa-catching nation in the world.^11,12^ The majority of the Hilsa catch (65%) comes from the marine waters of the northern Bay of Bengal, with the remaining 33% coming from the Meghna estuary system. The Padma and other inland riverine systems comprise only up 2% of overall Hilsa production (2%).^13^ Numerous investigations revealed that Bangladesh’s main rivers, including the Padma and Meghna, have distinct physicochemical properties^14–16^ that influence the growth rate and dominance of plankton populations,^13,16^ whereas estuarine phytoplankton is a significant source of food for Hilsa.

Interestingly, people of Bangladesh and West Bengal in India believe that Hilsa fish from the Padma River are tastier than those from other riverine systems. A recent study on Hilsa fish from two Indian river systems found that the better taste of Hilsa from the Padma River might be linked to higher levels of n3:n6 fatty acids and higher amounts of alanine, aspartic acid, glutamic acid, oleic acid, and palmitoleic acid.^17^

While Hilsa is an anadromous species, it migrates between marine and freshwater environments during its life cycle.^18^ This migration exposes the Hilsa fish to various environmental conditions, including variations in water salinity, diet, and pollution.^19,20^ According to De et al.,^17^ the lipid and amino acid profiles of Hilsa fish have changed dramatically across size groups and various habitats of Indian rivers and marine environments. Additionally, there have been significant variations in the proximate composition, mineral content, total protein content, and fatty acid content of two Tenualosa sp. from three distinct east coast areas of India.^10^ It has also been reported that the texture of fish muscle is strongly correlated with developmental stages and environmental factors, such as food habits.^21,22^

Regardless of its economic importance and popularity, Hilsa is notable for its possible allergy risk.^23,24^ According to a recent cross-sectional self-declaration survey of 970 people, Hilsa is one of the fish species that causes the greatest number of allergies in Bangladesh.^25^ Changes in the muscle proteome of Hilsa fish in response to different riverine habitats and developmental stages are therefore not surprising, which raises the question of whether these environmental factors may influence the amounts of PRV, the primary protein that causes fish allergies.^26,27^ More importantly, to the best of our knowledge, there is no report on the characterization of Hilsa PRV proteins. Therefore, this study aims to examine the PRV levels in young or small (also known as jatka) and adult (large) Hilsa fish in relation to the two main riverine systems in Bangladesh. Consequently, understanding the variations in PRV levels between different life stages of Hilsa fish will enhance our understanding of their ecological dynamics. This knowledge may also inform management strategies to ensure the sustainability of Hilsa populations in the face of environmental changes and fishing pressures. Therefore, further research into the structural and functional characteristics of Hilsa PRV proteins could provide important insights into their involvement in Hilsa fish biology and potential food safety consequences. The results of our study enhanced our understanding of fish allergies and the sustainability of Bangladesh’s Hilsa fishery, while also providing crucial information regarding the food safety of Hilsa fish.

## 2 Materials and Methods

### 2.1 Hilsa Fish Sample Collection

In Bangladesh, two different sizes of live Hilsa fish were collected from two major rivers, namely, Meghna and Padma. The Meghna (22°50′03.5″N, 90°20′06.5″E) and Padma (24°29′32.1″N, 88°18′17.6″E) rivers are located in the south and north side of the country, respectively (Figure 1A).

**Figure 1:**
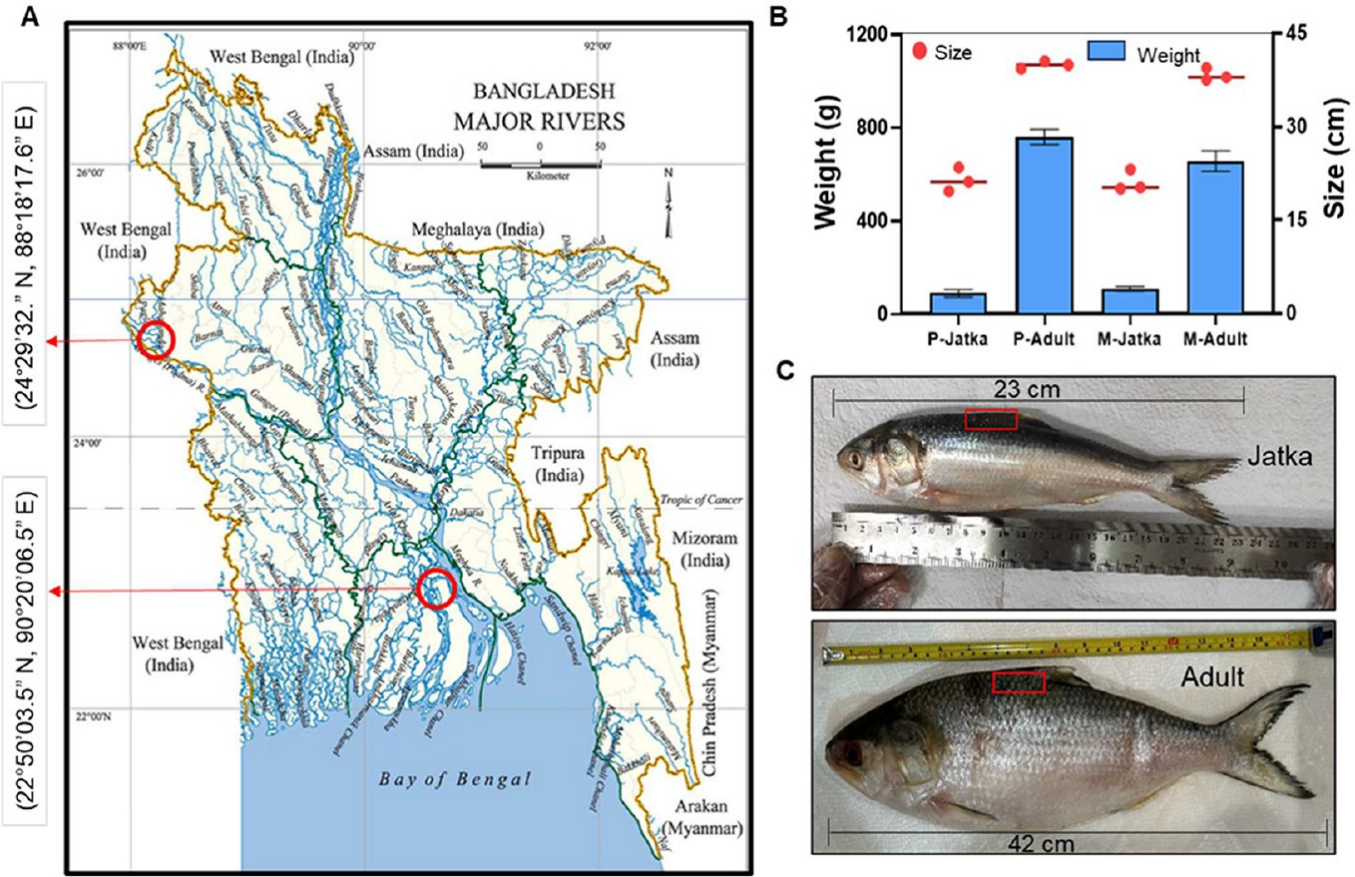
Hilsa fish collection and morphological characteristics. (A) Fresh Hilsa fish were caught from two main riverine systems, namely, Meghna (22°50′03.5″N, 90°20′06.5″E) and Padma (24°29′32.1″N, 88°18′17.6″E). Red circles in the map showing the sampling sites (geographic coordinates and place). The map was adopted from the national encyclopedia of Bangladesh (https://en.banglapedia.org/index.php/River). (B) Average (*n* = 3) size and weight of jatka (young/small) and adult (large) Hilsa fish were used for proteomic analysis. P and M indicate Padma and Meghna, respectively. (C) Muscle tissues were collected from the dorsal portion of each fish (marked by a red box) and were subjected to subsequent protein extraction and analysis.

Based on a recent study, female Hilsa from multiple populations in Bangladesh reach maturity (M50) at 31 cm total length.^28^ In this study, Hilsa fish having an average weight of 90.16–109.5 g and length of 21.2–21.46 cm are referred to as jatka (young/small), while those having an average weight of 658.26–761 g and length of 38.36–40 cm are classified as adult (large) (Figure 1B). It has been reported that generally jatkas are predominant in freshwater rivers, whereas adult fishes are primarily found in the estuary and marine water.^29^ For each Hilsa group, three fishes were subjected to subsequent proteomic analysis. Detailed procedures for fish sample collection have been previously reported.^30,31^ Briefly, live Hilsa fish were collected from the river. As Hilsa are extremely sensitive to stress, possess a fragile body structure, and have high oxygen demands due to their continuous swimming behavior, they die rapidly after removal from water. To preserve sample integrity and prevent spoilage, freshly caught fish were immediately transferred from the nets into insulated containers containing dry ice, and a low temperature was maintained throughout transportation to the laboratory. Fish samples were thawed and carefully cleaned to remove scales and surface mucus. Muscle tissue was carefully dissected from the dorsal region (Figure 1C), immediately frozen in liquid nitrogen, and subsequently stored at −80 °C until protein extraction and further analysis. All the required paperwork for animal ethics clearance and fieldwork was approved by the authority before the start of this study (ref. No.: KUAEC-2021/09/20).^31^

### 2.2 Muscle Protein Extraction and Proteomic Analysis

Muscle proteins were extracted using RIPA buffer (Thermo Scientific, Cat # 89901, USA) supplemented with protease inhibitors, as previously described in our Hilsa proteome study.^31^ Approximately 100 μg of total protein from each sample was subjected to in-solution digestion using Trypsin/LysC (Promega, cat. no. V5071, USA) at an enzyme-to-substrate ratio of 1:50, followed by incubation overnight at 37 °C in a rotator shaker. Digested peptides were desalted using C18 Sep-Pak cartridges (Waters, Milford, MA, USA), vacuum-dried, and reconstituted in 0.1% formic acid prior to LC–MS/MS analysis. Peptide separation was performed using a Dionex UltiMate 3000 UHPLC system (Thermo Fisher Scientific, USA) coupled to a Q Exactive HF-X mass spectrometer (Thermo Fisher Scientific, USA), following our recently published protocol with some modifications.^32^ Briefly, tryptic peptides (2 μg) were loaded onto a C18 analytical column (75 μm × 25 cm, 2 μm particle size) and separated using a 90 min gradient of 5–35% acetonitrile in 0.1% formic acid at a flow rate of 300 nL/min. The mass spectrometer was operated in positive-ion mode with a data-dependent acquisition strategy: full MS scans (*m*/*z* 350–1600) were acquired at 60,000 resolution, followed by Top15 MS/MS scans at 15,000 resolution using HCD fragmentation with a normalized collision energy (NCE) of 27. The AGC target values and maximum injection times were set according to our previous study.^31^

Raw data were subjected to Proteome Discoverer (PD) version 2.4 (Thermo Fisher Scientific, USA) and searched against the Hilsa protein sequence database described previously.^31^ Peptide-spectrum matches and proteins were filtered at a 1% false discovery rate (FDR) using the Percolator algorithm within Proteome Discoverer. Label-free quantification was performed using the Minora feature detection node, and proteins showing ≥ 1.5-fold change with *p*-value <0.05 were considered significantly altered.

### 2.3 Quantitation of Parvalbumins Using ELISA

Eleven different fish species, i.e., Atlantic salmon (*Salmo salar*), Atlantic cod (*Gadus morhua*), Mahi mahi (*Coryphaena hippurus*), Tilapia (*Pelmatolapia mariae*), Catfish (*Ictalurus punctatus*), Swai (*Pangasius bocourti*), Bighead carp (*Hypophthalmichthys nobilis*), Grass carp (*Ctenopharyngodon Idella*), and Bigmouth buffalo fish (*Ictiobus cyprinellus*), were collected from the Chinese fish market in Oklahoma City, USA. Similarly, frozen small (<500 g, *n* = 5) and large (>800 g, *n* = 9) Hilsa fish were collected from local Indian grocery stores located in Oklahoma City, USA. Frozen muscle tissue (1 g) was collected from the dorsal portion of each fish and stored at −80 °C until further use.^30^

PRVB protein quantitation was conducted using the Fish Parvalbumin ELISA Kit (ARG80797, Arigo, Taiwan). Recombinant mackerel (*Scomber japonicus*) parvalbumin-beta (Sco j 1, P59747) was used as a standard to generate calibration curves.^33^ All steps followed the vendor’s protocol with minor modifications. Briefly, 100 mg of fish was homogenized in 1.5 mL of extraction and dilution buffer using a Bead Mill 24 (*S* = 6.00, *C* = 01, *T* = 1:00, Fisherbrand, CA, USA). The mixture was then incubated at 60 °C for 15 min, followed by centrifugation at 2000g for 10 min to collect the supernatant. Subsequently, a 10-fold dilution of all fish samples was performed, followed by centrifugation at 7000*g* for 5 min to obtain particle-free solutions for the ELISA experiment. Finally, the optical density (OD) was read at 450 nm (reference OD 620 nm) within 30 min using a microplate reader (Synergy H1, BioTek, VT), and the protein concentration was quantified based on the standard parvalbumin using a logistic curve fit.

### 2.4 Multiple Reaction Monitoring (MRM) Analysis of Hilsa and Other Parvalbumin Proteins

A total of seven unique peptide sequences that correspond to the PRVs of six different fish species, i.e., the Hilsa, Tilapia, Catfish, Swai, Bighead carp, and Grass carp, were selected for a targeted proteomic analysis using a triple quadrupole mass spectrometer (TSQ Quantiva, Thermo Fisher Scientific, Germany) coupled with a nano UHPLC (Dionex 3000, Thermo Fisher Scientific, Germany).

To create a complex fish mixture, 10 μg of trypsin-digested peptides from each fish species were combined in a low protein-binding Eppendorf tube. Complex tryptic peptides (2 μg) were chromatographically separated using reverse-phase chromatography with acidified ACN (acetonitrile with 0.1% formic acid) and water (0.1% formic acid in LC–MS grade water) as the mobile phases. Separation of peptides was achieved with a total 60 min run with an analytical gradient of ACN from 5 to 35% in 30 min at a flow rate of 350 nL/min through an EasySpray (3 μm, 75 μm × 15 cm, ES900, Thermo Fisher Scientific, Germany) analytical column, followed by a quick increase to 95% ACN as a wash step, then equilibrated back to 5% ACN for the remaining 10 min.

For all of the peptides, the y-ions were selected for MRM analysis. The MRM method was developed using Skyline software version 24.1 (https://skyline.ms), an open-source tool for mass spectrometry analysis (Table S1). The parameters used for the quadrupole mass spectrometer instrument were as follows: positive-ion spray voltage, 2200 kV; ion transfer tube temperature, 350 °C; cycle time, 0.5 s; chromatographic peak width, 3.0 s; collision pressure, 1.5 mTorr; and Q1 and Q3 resolutions, 0.2 and 0.7 (fwhm), respectively. MS RAW files were further processed and analyzed by Skyline software (Ver. 24.1.0.199).

### 2.5 Bioinformatics Analysis

PRV sequences were downloaded from the WHO/IUIS Allergen Nomenclature Database (https://www.allergen.org/pubs.php). Multiple amino acid sequence alignment was performed using an open-source sequence alignment platform Clustal Omega (https://www.ebi.ac.uk/jdispatcher/msa/clustalo), and a heatmap on percent (%) identity was generated using Python. Phylogenetic analysis was performed by using the neighbor-joining (NJ) method through MEGA V. 11 ^34^ and visualized by iTOL V. 6.^35^ Bootstrap test percentages (≥60%) of 10,000 replicates are shown next to the branches.^36^ PCA and Volcano plots were further generated and visualized by SRplot,^37^ an open-source online bioinformatic platform. For PCA, 95% confidence ellipses were used to cluster the data points from each group. Volcano plots were generated using a fold-change threshold of ≥1.5 and a *p*-value <0.05. Histograms were generated using a Python script (https://github.com/tingying-he/KaiVis/blob/main/protein-rank-bar-chart-with-highlights.ipynb). Bar and Violin plots were generated with GraphPad Prism 7.0. The Immune Epitope Database (IEDB) (http://tools.iedb.org/bcell/) was used to predict B-cell epitopes in accordance with the Kolaskar and Tongaonkar Antigenicity platform. The 3D structures of PRV variants were predicted using AlphaFold2,^38^ and structural visualization and analysis were performed using PyMOL.^39^

## 3 Results

### 3.1 Identification of Parvalbumin Isoforms in Hilsa Muscle Tissue

A total of 4448 unique peptides corresponding to 646 unique protein groups were successfully identified and quantified from Hilsa muscle samples (Tables S2 and S3). Among these, three proteins were identified as parvalbumins (PRVs) with high sequence coverage ranging between 72 and 74%. Among these three PRVs, the accession T_ilisha_TRINITY_DN10771_c0_g2_i1.p1 was annotated in the database as parvalbumin-beta (PRVB), whereas T_ilisha_TRINITY_DN37439_c0_g1_i3.p1 and T_ilisha_TRINITY_DN10771_c0_g1_i1.p3 were both annotated as parvalbumin-alpha (PRVA).

Each Hilsa PRV was uniquely identified by at least four distinct peptides, providing high confidence for the identification of multiple PRV isoform amino acid sequences in Hilsa muscle tissue (Figure 2A and Table S4). The three Hilsa PRVs have extremely different N-terminal sequences, and we were able to identify these distinct peptides using our discovery proteomic approach (Figure 2B).

**Figure 2:**
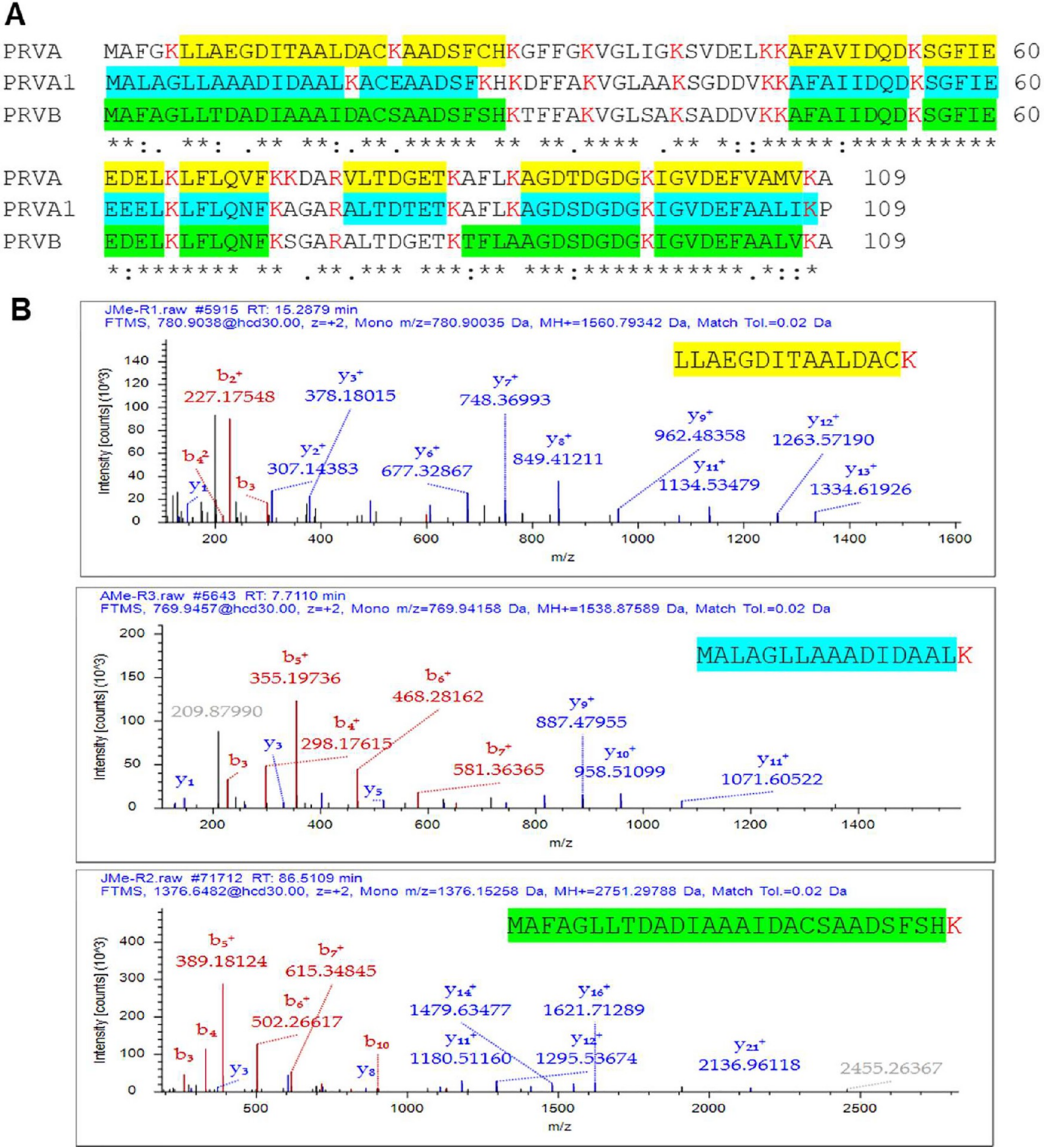
LC–MS/MS proteomic characteristics of Hilsa parvalbumin isoforms. (A) Multiple amino acid sequence alignment of three PRV isoforms of Hilsa fish. Peptide sequences labeled as yellow (PRVA, parvalbumin-alpha), cyan (PRVA1, parvalbumin-alpha1), and green (PRVB, parvalbumin-beta) were identified by LC–MS/MS analysis. (B) LC–MS/MS fragmentation three distinct peptides correspond to N-terminal of PRVA (yellow), PRVA1 (cyan), and PRVB (green) isoforms.

To better understand the phylogenetic relationship of these three Hilsa PRVs, we further conducted a phylogenetic analysis of over 170 parvalbumin sequences from 10 distinct PRV gene families (*pvalb1–10*) in bony fish and α-parvalbumins from other vertebrates, including human, mouse, shark, frog, chicken, and crocodile, to better understand the diversity and evolutionary distinctions of Hilsa PRV isoforms.^40^ Our findings revealed that Hilsa PRVB is closely related to the *pvalb1* and *pvalb2* gene clusters, while PRVA and PRVA1 exhibited closer evolutionary relationships with the *pvalb4* and *pvalb3* groups, respectively (Figure 3). Therefore, based on the phylogenetic relationships, we have renamed PRVB as “Hilsa PVRB1,” PRVA as “Hilsa PRVB4,” and PRVA1 as “Hilsa PRVB3,” corresponding to their close homology with *Clupea harengus* *pvalb*4 and *pvalb*3, respectively. As emphasized by Dijkstra et al.,^40,41^ parvalbumin proteins should be named according to their phylogenetic relationships. Thus, the revised nomenclature of the Hilsa PRVAs is well-supported, and all identified Hilsa PRVs can appropriately be considered to be PRVB isoforms.

**Figure 3:**
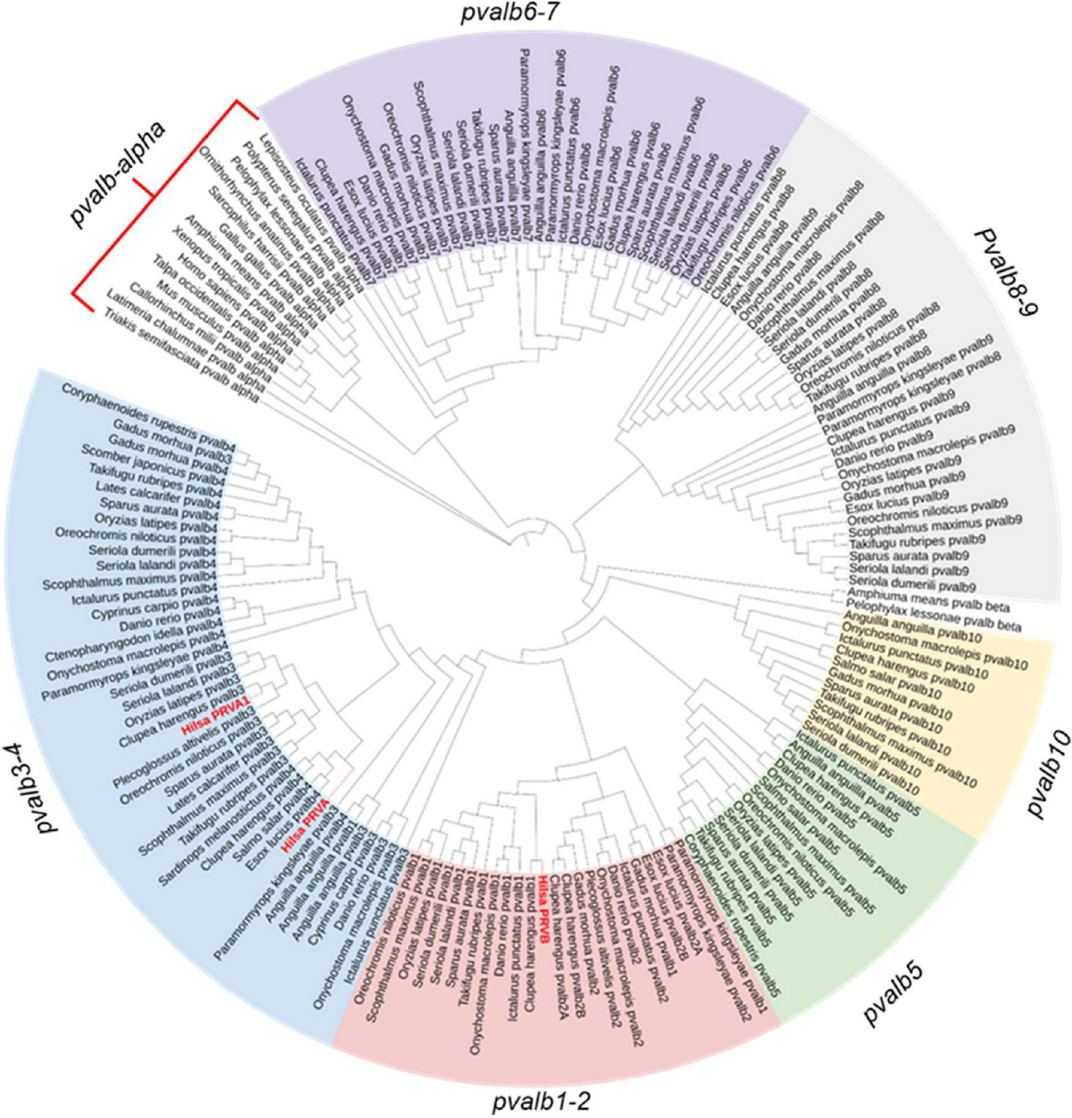
Phylogenetic analysis shows the relationship of Hilsa parvalbumins with the entire fish and other species parvalbumins. The phylogenetic tree was constructed using 170 PRVs in total, including three Hilsa PRVs denoted by red text, by the open-source platform NGPhylogeny.fr (https://ngphylogeny.fr). The PRV sequences are curated from the previously published report that contained all bony fish PRVs and α-parvalbumins from other vertebrates.^40^

As expected, Hilsa PRV isoforms exhibit a lower sequence identity (<61%) with the known PRVAs (Figure 4A), while the known PRVAs from frogs, chickens, crocodiles, and other vertebrates cluster together (Figure 3). This finding implies that Hilsa muscle PRVs are distinct from the other vertebrate PRVAs and have evolved a close relationship to the fish *pvalb1–4* family. Furthermore, an in silico analysis of Hilsa PRVs’ B-cell epitope map and scores revealed that they were very similar to the well-characterized PRVBs of a common carp PRBV allergen (cypc1.02) (Figure S1).

**Figure 4:**
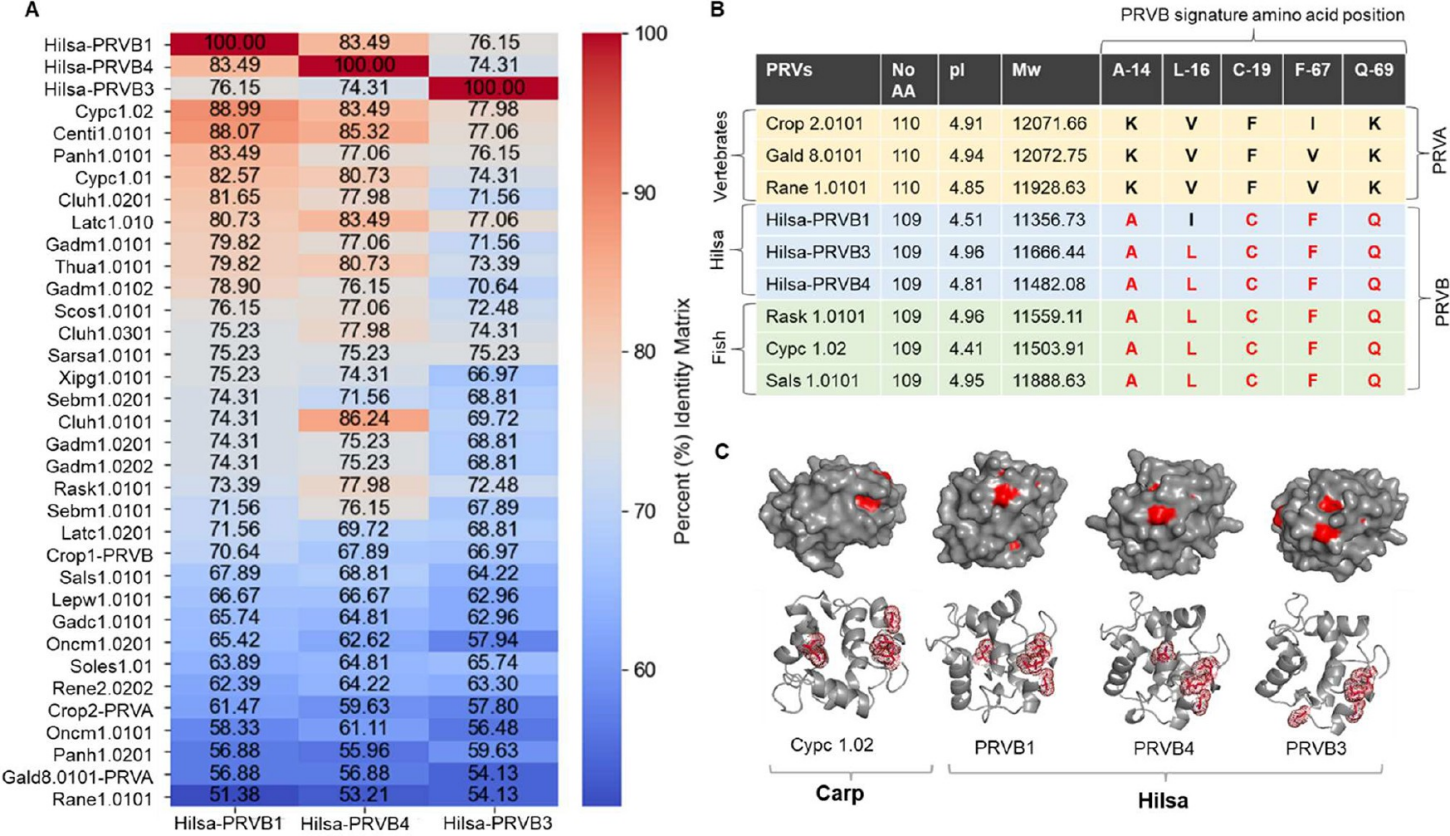
Evolutionary relationship between Hilsa parvalbumins and all WHO/IUIS-registered parvalbumins. (A) Heat map showing the amino acid sequence identities (%) of all WHO/IUIS-registered parvalbumins and Hilsa PRVs. The sequence identities were calculated using Multiple Sequence Alignment in Clustal Omega (EMBL-EBI). (B) Comparative table shows the theoretical pI/Mw and the signature amino acid position of Hilsa PRVs compared to three well-characterized PRVB and PRVA. (C) Structural comparison of PRVB (Cypc1.02, Common carp) with the three Hilsa PRVs (PRVB1, PRVB3, and PRVB4). The 3D structures of Hilsa PRVs were predicted using AlphaFold2 and visualized in PyMOL. Red regions represent predicted B-cell epitope binding sites.

A multiple sequence alignment with WHO/IUIS-registered parvalbumin protein sequences revealed that all three Hilsa PRVs have 74–83% sequence identity (Figure 4A), despite the fact that Hilsa PRVB has a high percentage of sequence similarity (80–89%) with freshwater carps, catfish, and herring PRVBs (Figure 4A). However, Hilsa PRVA isoforms have a higher sequence identity (>70%) with other fish species belonging to the Clupeiformes, including Pacific pilchard (Sarsa1.0101) and Atlantic herring (Cluh1.0101, Cluh1.0201, and Cluh1.0301). A side-by-side amino acid sequence analysis of Hilsa PRVs with three well-characterized PRVA and PRVB revealed no significant differences in the theoretical pI and Mw of all three Hilsa PRV isoforms from those of other PRVs. However, the amino acid positions of the known allergen PRVB attributes are highly conserved for all three Hilsa PRVs (Figure 4B). To further investigate structural conservation and allergenic potential, the 3D structures of all three Hilsa PRVs were predicted using AlphaFold2 and compared with the well-characterized PRVB from common carp (Cypc1.02) (Figure 4C). All Hilsa PRVs exhibited highly similar 3D folds to the reference structure, particularly in the EF-hand domains typical of calcium-binding parvalbumins.^40^ The predicted IgE-binding epitope sites, visualized in red, were found at nearly identical spatial positions across all structures, with comparable confidence scores. This structural overlap suggests that Hilsa PRVs may share similar immunogenic profiles with known allergenic parvalbumins.

### 3.2 Relative Abundance of Hilsa PRV Isoforms in Muscle Tissue

We further performed a relative abundance–based quantitative analysis to assess the abundance patterns of the three PRV isoforms in comparison with other proteins identified in Hilsa muscle tissue across different developmental stages and geographic locations. The results showed that PRVB3 and PRVB4 are consistently among the most abundant proteins in Hilsa muscle tissue, surpassing PRVB1 irrespective of developmental stage or geographic distribution (Figure 5). However, they exhibited relatively higher abundance in the jatka groups comparative to adult Hilsa.

**Figure 5:**
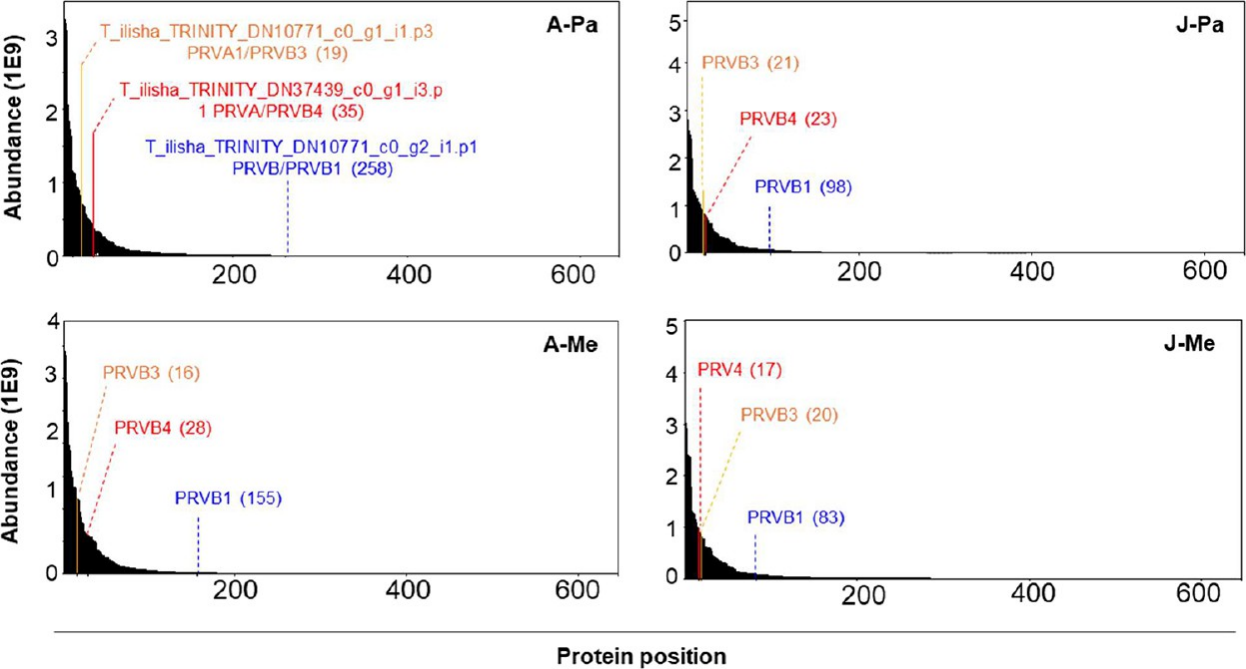
Relative quantitation of PRV isoforms in Hilsa muscle tissue. The histogram shows the relative abundance of Hilsa PRVB1, PRVB3, and PRVB4 in different Hilsa samples collected from two different riverine systems.

### 3.3 Comparative Proteome Analysis of Adult and Jatka Hilsa Collected from the Padma and Meghna Rivers

The label-free comparative proteome analysis of total Hilsa muscle protein abundance shows tight clustering within the two adults (APa and AMe) and two jatka groups (JPa and JMe). However, there is a significant difference between the two sizes (adult and jatka; Figure 6A). Similarly, a heat map analysis of the total number of unique proteins further demonstrates the differential abundance between the size groups (Figure 6B). Regardless of the two distinct riverine systems, volcano plot analysis also showed that only PRVB1, out of the three PRVs found in this study, demonstrated significant alteration (at least 1.5-fold with a *p*-value < 0.05) between the adult and jatka fish (Figure 6C, D). The bar diagrams show the overall abundance of the three PRVs compared with the Hilsa samples (Figure 6E–G).

**Figure 6:**
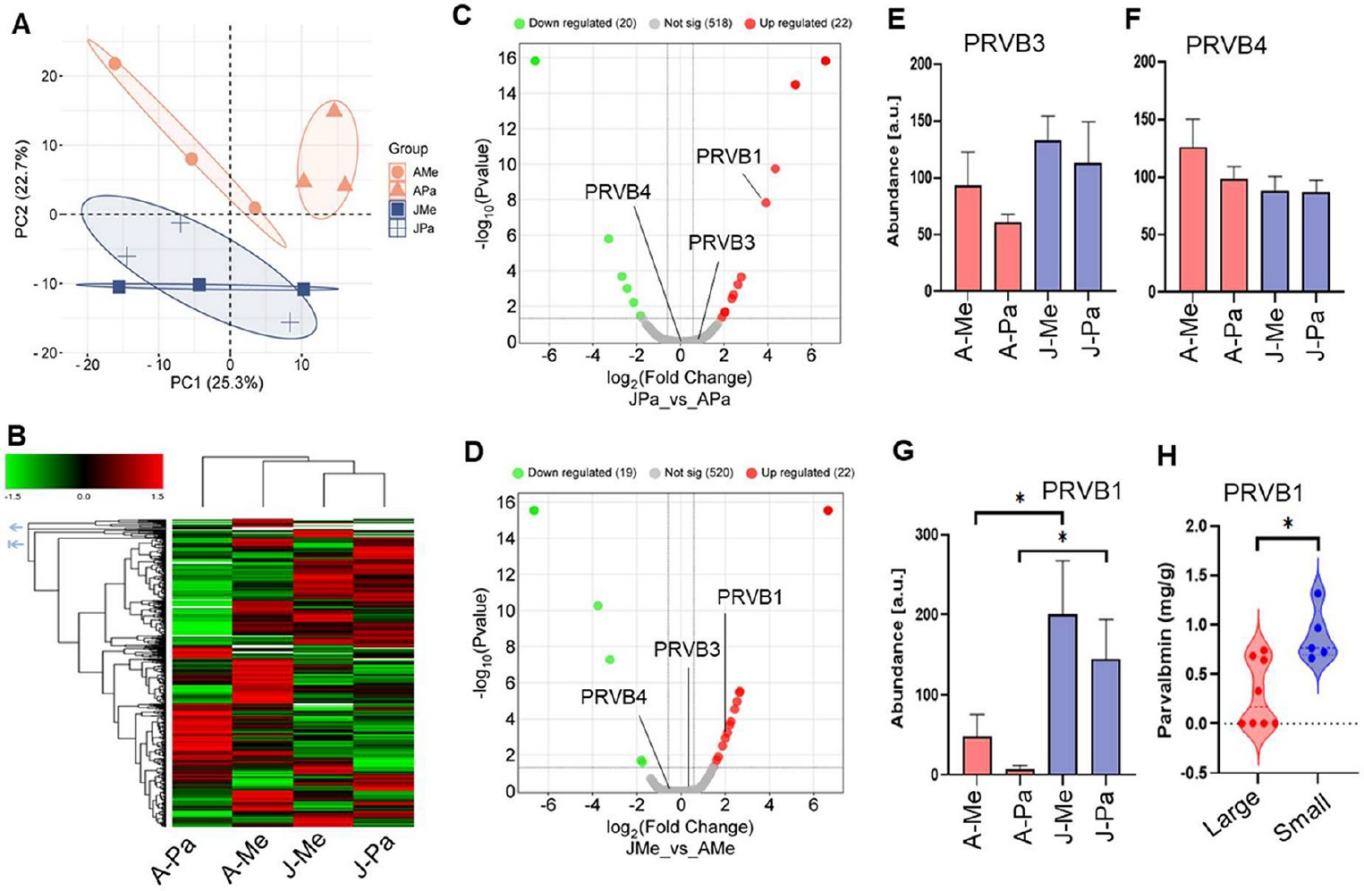
Comparative proteome profiles of adult and jatka Hilsa fish from the Meghna and Padma rivers. (A) Principal component analysis (PCA) of normalized total protein abundance (peak area) of adult (A) and jatka (J) Hilsa fish muscle samples collected from the Meghna (Me) and Padma (Pa) rivers. The PCA plot clearly separates the adult (AMe and APa) and jatka groups (JMe and JPa). (B) Heatmap illustrates the differential expression of grouped abundance 646 proteins identified and quantified across the four groups: AMe, APa, JMe, and JPa. The color gradient reflects the relative increase (red) or decrease (green) in protein abundance, highlighting significant distinctions between the adult and jatka groups. (C, D) Volcano plots showing the differential protein expression between JPa vs APa (C) and JMe vs AMe (D) groups with significantly (at least 1.5-fold and adjusted *p*-value > 0.05) upregulated (red) and downregulated (green) proteins. Gray dots represent proteins that are not statistically significant between the groups. (E–G) Bar diagrams represent PRVB3 (E), PRVB4 (F), and PRVB1 (G) protein abundance in four Hilsa samples quantified by label-free proteomic analysis. Asterisk over the bars in PRVB indicating statistical significance between the size (adult and jatka) of Hilsa fish samples (G). (H) Violin plot shows the relative PRVB contents measured by ELISA in adult (*n* = 9) and young (*n* = 5) Hilsa muscle tissues.

Although there was no discernible difference in PRVs between the two riverine systems, our proteomic study demonstrated significant differences in PRVB abundance between the adult and jatka Hilsa groups. Therefore, we further intend to cross-validate the proteomic results by measuring the PRVB content in Hilsa fish samples using an independent sandwich ELISA assay, utilizing an unbiased Hilsa sample pool collected from the local fish market. Relative quantitative analysis revealed that smaller Hilsa fish (<500 g) contained about 1.32 mg/g of PRVB, while larger Hilsa fish (>800 g) contained 0.62 mg/g (Figure 6H).

### 3.4 Relative Quantitation of Parvalbumin-Beta in Hilsa and Other Fish Species

To better understand the relative PRVB content of Hilsa fish compared to those of other commonly consumed freshwater and saltwater fish species, we measured the PRVB of 10 fish species using the ELISA assay. As anticipated, fish-to-fish variation in PRVB content was substantial (Figure 7).

**Figure 7:**
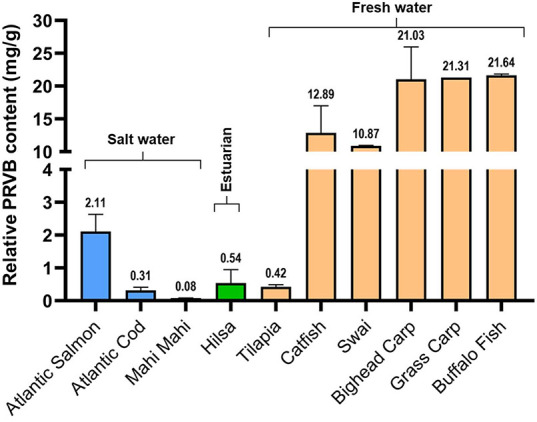
Relative parvalbumin-beta (PRVB) contents in the dorsal muscle tissues from 10 species of fish. Three biological samples from each of the other fish species and 14 Hilsa fish ranging between 500 and 1000 g were used for ELISA analysis. Each bar represents the average (±SD) and the actual value indicated above the bars.

Compared to saltwater fish species, freshwater fish had much greater levels of PRVB. While the PRVB contents of three marine fish species (Atlantic salmon, Atlantic cod, and Mahi mahi) were extremely low (0.08–2.11 mg/g), PRVB contents were much higher (4.65–21.64 mg/g) in seven freshwater fish species (Catfish, Swai, Bighead carp, Grass carp, and Buffalo fish). Interestingly, PRVB content was extremely low in the estuarine fish Hilsa and the most cultured fish Tilapia, at 0.54 and 0.42 mg/g, respectively (Figure 7). Overall, except for Tilapia, the PRVB concentration of Hilsa fish was 40 times lower than that of other highly consumed Asian freshwater fish species.

### 3.5 Hilsa Parvalbumin for Targeted Analysis of Food Safety and Authenticity

A targeted proteomics experiment utilizing multiple reaction monitoring (MRM) was conducted to identify the unique peptides that correlate to PRVs in six frequently ingested freshwater fish species: the Swai, Grass carp, Hilsa, Catfish, Tilapia, and Bighead carp. The Hilsa peptides IGVDEFAALVK and IGVDEFVAMVK, which correspond to PRVB1 and PRVB3, respectively, were successfully identified by our discovery proteomics approach (Table S4).

Unique PRV peptide sequences for all other fish species were identified by using Skyline software. Except for the Hilsa PRVB1 peptide KIGVDEFAALVK (Yellow, Figure 8A), shared with Tilapia, the PRV peptide sequences unique to each fish species are highlighted in green (Figure 8A). A trypsin-digested peptide mixture of six fish species was analyzed by LC–MS/MS using a triple quadrupole mass spectrometer. The results reveal distinct chromatographic separation with unique retention times of each PRV peptide, confirming the specificity of the fish species (Figure 8B). Furthermore, the coelution of multiple transition ions under the same retention time for each PRV peptide confirms these peptides’ optimal selection and reliability for the future development of MRM-based techniques to identify and quantify PRV isoforms from any complex fish matrix (Figure 8C).

**Figure 8:**
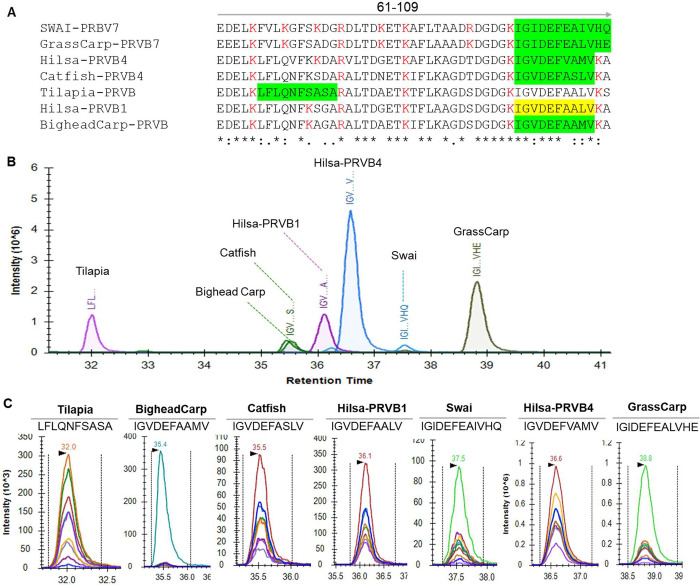
Selection and identification of ideal unique parvalbumin peptides of six different fish species using multiple reaction monitoring (MRM) for targeted proteomics analysis. (A) Multiple amino acid sequence analysis of Tilapia, Bighead carp, Catfish, Swai, Grass carp, and Hilsa shows the unique (green) and shared peptides in the C-terminal of PRVs. (B) Chromatogram representing the seven peptides corresponding to PRVs of six different fish species. (C) Transition peaks on Skyline for precursor peptides LFLQNFSASA, IGVDEFAAMV, IGVDEFASLVKA, IGIDEFEAALV, IGVDEFVAMV, IGIDEFEAIVHQ, and IGIDEFEALVHE. Each color represents a different transition ion (i.e., precursor peptide/fragment ion pair).

## 4 Discussion

Fish is one of the “big nine” sources of food allergies. Since PRVs are the primary muscle proteins that cause allergic responses, risking food safety and human health worldwide. Therefore, identifying, characterizing, and quantifying the PRV proteins in any species of fish is the first step in studying food safety and fish allergies.^42^ Like many other fish species, Hilsa has also been shown to have IgE-mediated hypersensitivity reactions.^23,24^ More importantly, a recent survey on food allergy sensitivities found that Hilsa is one of the top three foods that cause allergic responses in Bangladeshi population, and that over 30% of Northern Indians patients in the age group 5–40 years experienced allergic reaction to Hilsa fish.^43^

These earlier investigations clearly showed that Hilsa is allergic, similar to many other bony fish species, with PRVB being the primary source triggering allergic reactions.^42,44–47^ However, there are currently no comprehensive reports on Hilsa parvalbumins, the primary known cause of fish allergies. Thus, a deeper comprehension of the protein sequences and isoforms and comparative quantitative analysis of Hilsa parvalbumins in muscle tissue may result in better diagnostic tools and possible therapeutic interventions. Furthermore, this knowledge may help develop guidelines for safe consumption and management of Hilsa-related allergies among affected populations.

The LC–MS/MS-based proteomic approach is one of the advanced analytical platforms commonly used for identification and characterization of fish allergens like parvalbumins. Thus, so far, only two proteomics studies on Hilsa fish that have been published focus on how muscle tissue^31^ and serum^48^ respond to different developmental stages. Additionally, a metabolomic profile of Hilsa fish revealed that the nutritional composition of the fish is significantly altered by fecundity, geographic distribution, and growth and developmental phases.^49,50^ However, to the best of our knowledge, no study has investigated how allergen proteins respond to the geographical location and developmental stage of Hilsa fish. In this work, we use LC–MS/MS techniques to characterize PRVs, the primary allergen of Hilsa fish, in relation to their geographic distribution and developmental stage.

For the first time, in this report, we revealed the actual peptide sequences of three PRV isoforms found in Hilsa muscle tissue using a discovery proteomic approach. Phylogenetic analysis of Hilsa PRVs with the entire bony fish PRVBs and nonfish PRVAs further revealed that all three Hilsa PRVs are members of the teleost *pvalb1–4* gene family (Figure 3). Remarkably, the *pvalb1–4* gene family, which is collectively referred to as allergens, was significantly expressed in muscle and had high sequence similarity (>85%) to each other.^3,40^ Furthermore, comparative amino acid sequence analysis of Hilsa PRVs with WHO/IUIS-recognized PRV allergen sequences revealed that Hilsa PRVB shares close similarity with PRVBs from freshwater fish species, including Common carp, Grass carp, and Catfish. Interestingly, among the three Hilsa PRVs, the sequence identity was between 72 and 74% (Figure 4A). Bony fish have been found to contain multiple copies of parvalbumin genes (*pvalb*) (1–10), which vary significantly between species.^40^ For instance, rainbow trout and barramundi have lower intraspecies amino acid sequence identity (<68%) of PRVs, indicating the diversity of allergenic parvalbumins.^42^

On the other hand, Hilsa PRVs showed lower sequence identity (<61%) with well-characterized PRVAs of chicken, frog, and crocodile (Figure 3). Nonetheless, the amino acid locations of the PRVB characteristics are highly conserved, and the three Hilsa PRVs have an identical number of amino acids (Figure 4B). In addition to sequence-level analysis, structural modeling of Hilsa PRVs provided deeper insights into their potential allergenicity. The predicted 3D structures of the three Hilsa isoforms were highly conserved and closely resembled the well-characterized PRVB structure from common carp (Cypc1.02) (Figure 4C), with preserved EF-hand motifs and consistent folding patterns.^40^ Notably, surface-mapped IgE-binding epitopes were spatially conserved across all structures, suggesting that structural features contributing to allergenicity are maintained despite isoform differences. These findings underscore the importance of combining sequence-, structural-, and epitope-level analyses to comprehensively assess the allergenic potential of fish parvalbumins.

Furthermore, a comparison of Hilsa PRVs’ B-cell epitope mapping and epitope scores revealed that they were very comparable to the well-characterized PRVB of common carp (cypc1.02) (Figure S1).These findings provide robust support for the distinct identity and evolutionary separation of the three Hilsa PRV isoforms and align with recent reports highlighting the complexity, microheterogeneity, and lineage diversification within the parvalbumin gene family in teleost fish.^40^ However, further studies are necessary to elucidate the specific biological implications of these Hilsa PRVs in the context of allergenicity.

Measurement of the PRVB level is the first step in identifying the allergy sensitivity of a fish species.^42^ Despite differences between the two riverine habitats, label-free quantitative proteomic analysis reveals that relative PRVB abundance was significantly lower in adult Hilsa compared to jatka fish, while PRVA levels remained constant (Figure 6C–G). We acknowledge that the sample size (*n* = 3) per group may limit a study that draws population-level implications; however, it is important to keep in mind that jatka fishing is illegal in Bangladesh. Thus, collecting a higher number of jatka samples is a further major challenge. However, our investigation demonstrated that jatka (a total of six fish from two distinct places) exhibited a pattern of PRVB abundance similar to that of the adult, supporting the steady trend of PRVB levels in smaller Hilsa fish. We further cross-validate this finding using an alternative targeted protein quantitation method mostly known as ELISA using unbiased Hilsa fish samples (large and small) that were collected from the local market (Figure 6H). Although the specific riverine environment is unknown, the package label states that these Hilsa fish samples are from Bangladesh. We acknowledge that the development of a species-specific ELISA for Hilsa would enable more accurate and direct quantification of PRVB isoforms. Nevertheless, the principal objective of the present study was not to design or optimize an ELISA assay but rather to employ it as a complementary analytical tool to corroborate MS-based findings. According to the vendor’s documentation, the commercial ELISA kit used in this study is designed to detect parvalbumin (PRVB) across multiple fish species, including herring (*Clupea harengus*) and mackerel. Phylogenetic analysis further revealed that Hilsa PRVB1 shares a close evolutionary relationship with *C. harengus* *pvalb1* (Figure 3). The use of a mackerel-derived standard for cross-species parvalbumin detection is well-supported in the literature. For instance, Shibahara et al.^51^ developed a sandwich ELISA using antibodies raised against Pacific mackerel parvalbumin, reporting 22.6–99% cross-reactivity with parvalbumins from other fish species such as eel, horse mackerel, sea bream, and tuna. Similarly, Dong and Raghavan^52^ utilized the same commercial kit calibrated with recombinant mackerel PRVB to quantify parvalbumin in Atlantic cod, demonstrating the cross-species validity of this approach. Therefore, the ELISA results obtained in this study are both consistent and biologically meaningful, reinforcing the robustness and reliability of MS-based findings.

Regardless of sample origin and quantification methods used to determine the PRVB level, these findings collectively imply that Hilsa PRVB is regulated by developmental stages, and that Bangladesh’s riverine environment has little effect on PRVB. This developmental regulation of PRVB may be associated with increased muscle activity or calcium-binding demand during early growth stages. Studies on migratory and fast-swimming fish have shown that juvenile fish exhibit greater expression of parvalbumin in white (fast-twitch) muscle fibers, which are linked to rapid swimming and escape responses.^53,54^ Despite being in a more metabolically active life stage, younger or jatka Hilsa fish may have greater PRVB levels as a physiological response to their swimming technique and the makeup of their muscle fibers. These findings are consistent with previous studies of the protein and lipid profiles of Hilsa fish.^17,31,48^ Due to their more significant movement activity and less dark muscle in the dorsal muscle region, smaller Hilsa fish may have higher levels of PRVB than larger fish.^6,44,53^ Future research on tissue-specific expression could further elucidate the mechanisms behind these variations and their implications for fish allergy management.

A common phenomenon strongly linked to allergic reactions to fish is the variation of PRVB levels in various fish species.^2,42,44,54–56^ However, the profiling of allergens in the great majority (>32 K) of fish species has never been reported.^57^ This gap in research highlights the need for comprehensive studies that can identify and characterize these allergens. By increasing our understanding of the allergenic profiles of various fish species, we can better inform public health policies and guide individuals with fish allergies in making safer dietary choices. In this regard, we also compare the levels of PRVB in Hilsa fish with those in other commonly consumed freshwater and multiple known marine fish species (Figure 7). Commercial ELISA kits are known to have limitations in species-specific detection and typically provide relative, rather than absolute, quantification. Cross-reactivity issues have also been documented.^58^ Our results align with those reported by Wai et al.,^59^ who employed SDS-PAGE and PARV-19 binding assays. Specifically, the relative distribution of PRVB between cod and salmon in our study mirrors the patterns observed by Sharp et al. using antisalmon and antipilchard antibodies. These findings indicate that despite interspecies variability in antibody-binding efficiency, generic PRVB ELISAs remain valuable for relative allergen detection and quantification. Accordingly, while this ELISA approach is appropriate for comparative analyses, its limitations in achieving absolute quantification should be acknowledged.

Regardless, our findings showed that relative PRVB level in Hilsa fish is more than 40 times lower than that of fish species in the carp family (Common carp, Buffalo fish, and Grass carp) and more than 20 times lower than catfish-like fishes (Catfish, Swai). However, the muscle tissue Hilsa PRVB levels (mg/g) are comparable to those of well-known marine fish species, such as Cod, Salmon, and Mahi Mahi. Our findings are consistent with earlier studies showing that freshwater fishes had higher levels of PRVB than marine or migratory fish species.^53^ Although Hilsa exhibited relatively lower PRVB levels compared with other freshwater species, this does not necessarily translate into reduced clinical allergenicity. Previous surveys in Bangladesh and India consistently report Hilsa as one of the most common allergenic fish. This apparent contradiction may be explained by multiple factors: (i) very high regional consumption of Hilsa, which increases exposure risk, (ii) lack of comparative data with other fish species, (iii) extensive cross-reactivity of PRVB epitopes across fish species, and (iv) preparation methods (e.g., cooking, processing) that may influence protein stability and allergen presentation. It has been reported that patients with parvalbumin-specific IgE alone often tolerate low-PRVB fish, such as swordfish or tuna.^55,60^ Therefore, discovering various low-parvalbumin fish species could offer new dietary choices for individuals with moderate to high threshold dose reactivity.^61^ Therefore, our comparative quantitative results should be interpreted as indicators of intrinsic allergen burden, while the real-world allergy risk is shaped by broader immunological and cultural contexts. Nonetheless, our proteomics data independently confirmed the presence of PRVB isoforms in Hilsa, supporting the validity of the comparative results. We emphasize that these ELISA-based comparisons should be interpreted as semiquantitative indicators of relative allergen presence across species rather than absolute measurements. Development of a Hilsa-specific PRVB assay in the future would further strengthen these findings.

Given its allergenic features, presence of fish in foods is almost globally subject to mandatory labeling.^62^ Notwithstanding, differences in allergen burden have a crucial impact on the allergenicity and tolerability of distinct fish species. Patients with singly PRVB-specific IgE alone often tolerate low-PRVB fish, such as swordfish or tuna.^55,60^ Therefore, evolving labeling from the simple declaration of the presence of fish to a process including the fish species will significantly improve food safety and authenticity. With this aim, unique PRV peptides have been effectively exploited as selective and specific markers of fish allergens.^63–66^ In addition to identifying major fish allergens and verifying fish products, a single peptide marker can be used to develop alternative LC–MS-based assays, such as AQUA (Absolute Quantification) MRM technology, to measure allergen levels in a complex combination.^67–70^ Here, using a targeted proteomic approach, two distinct peptides that correspond to Hilsa PRVs were further validated as potential Hilsa markers from a freshwater fish mixture comprising Grass carp, Bighead carp, Swai, Catfish, Tilapia, and Hilsa meat (Figure 8). Thus, the identification of isoform- and species-specific PRV peptides in Hilsa using targeted proteome analysis not only advances our understanding of the unique PRV peptide patterns associated with each fish species but also offers practical value for fish allergen detection. Furthermore, these peptides can be used to develop targeted LC–MS/MS or immunoassay-based tools for authentication and differentiation of seafood products for a variety of commercial and culinary applications.^71–74^ These findings further highlight the potential of LC–MS/MS as a trustworthy instrument for assessing the quality and authenticity of fish in the seafood sector.

In summary, for the first time, we define the PRVs of the Hilsa fish, the most economically important fish species in South Asian countries, particularly Bangladesh. We defined the amino acid sequences of three distinct PRVs in Hilsa muscle tissue. Comparative evolutionary study with all known PRV sequences revealed that all three Hilsa PRVs are closely related to the well-preserved allergic *pvabl1–4* gene family. Quantitative investigation shows that smaller fish have PRVB levels higher than those of larger fish, and Hilsa PRVB levels are 20–40 times lower than those of other popular freshwater fish species. The discovery of distinct PRV peptides validates the versatility of the LC–MS/MS proteomics technique in characterizing allergen proteins in fish species. Unique Hilsa PRV peptides not only provide a dependable platform for detecting and quantifying the Hilsa samples in processed fish products but also offer great potential for assessing food safety and lowering the risks of allergen contamination in packaged fish consumed globally. Since Hilsa has a lower PRVB than other popular freshwater fish species, incorporating Hilsa fish in diets may offer a sustainable way for individuals with PRVB-sensitive allergies to reduce allergy-related risks. Furthermore, the management of Hilsa fisheries will significantly benefit from the comparative quantification of PRVB levels in Hilsa fish to select the right size of fish.

## Supplementary Material











## Data Availability

All LC–MS/MS RAW files and results files can be found at the MassIVE database (https://massive.ucsd.edu) under the following accession MSV000096794.

## Notes

In silico allergen potential of Hilsa parvalbumins; (A) the amino acid sequence alignment of Hilsa PRVs with well-characterized common carp PRVB allergens Cycp 1.02, red color amino acid in the sequence represents the potential epitopes, the red box shows the parvalbumin-beta corresponding amino acids. B–C, the epitope region identified by IEDB (http://tools.iedb.org/bcell/) following Kolaskar and Tongaonkar method (PDF)
Detailed characteristics of parvalbumin peptides used for MRM analysis (XLSX)
List of peptides identified by LC–MS/MS analysis (XLSX)
List of Hilsa proteins identified by using LC–MS/MS analysis (XLSX)
Parvalbumin peptide sequences of Hilsa fish identified by LC–MS/MS analysis (XLSX)
