# Supplementary material for: Proteomic Characterization of Major Fish Allergy Responsive Protein Parvalbumins in Hilsa (Tenualosa ilisha): A Commercially Important Fish in Southeast Asia
Source: J Agric Food Chem. 2025 Nov 17;73(47):30551–64. doi: 10.1021/acs.jafc.5c04335 (PMC12670419; doi:10.1021/acs.jafc.5c04335)
Supplement: Supplementary file 1 [file jf5c04335_si_001.pdf]

# Supporting Information

**Proteomic characterization of major fish allergy responsive protein parvalbumins in Hilsa (Tenualosa ilisha): A commercially important fish in Southeast Asia**

Nazma Shaheen[1], Zongkai Peng[2], Amit Singh[2], Asfia Wahab[3], Maria Gasset[4], Md Hafizul Islam[1], Oumma Halima[1], Aleena F Ali[5], Salmaan M Shah[5], Malek Salkini[5], Saaim Saleemi[5], Abdullah F Mallah[5], Ayan Khan[6], Sohaib Mesiya[6], Morshed Khandaker[7], Aayan Zarif[8], Akbar Ali[10], Amir Samour[10], John W Peters[2], Zhibo Yang[2,9], Nagib Ahsan [2, 10, *]

[1] Institute of Nutrition and Food Science, University of Dhaka, Dhaka 1000, Bangladesh

[2] Department of Chemistry and Biochemistry, The University of Oklahoma, Norman, OK 73019, USA

[3] Department of Biology, University of York, York YO10 5DD, UK

[4] Institute of Physical-Chemistry Blas Cabrera, Spanish National Research Council, Madrid 28006, Spain

[5] Dodge Family College of Arts and Sciences, University of Oklahoma, Norman, OK 73019, USA

[6] Department of Biology, University of Central Oklahoma, Edmond, OK 73034, USA

[7] Nanobiology Laboratory, School of Engineering, University of Central Oklahoma, Edmond, OK 73034, USA.

[8] Mercy School Institute, Edmond, OK 73013, USA

[9] Department of Biochemistry and Physiology, The University of Oklahoma Health Sciences Center, Oklahoma City, OK 731034, USA

[10] Mass Spectrometry, Proteomics and Metabolomics Core Facility, Stephenson Life Sciences Research Center, The University of Oklahoma, Norman, OK 73019, USA

*** Corresponding author:**

Nagib Ahsan, PhD
Department of Chemistry and Biochemistry
The University of Oklahoma, Norman 73019, OK, USA
Email: nahsan@ou.edu
Tel: +1 405-325-2852

# Figure S1.

A

```
PRVA        MAFGKLLAEGDITAALDACKAADSFCHKGFFGKVGLIGKSVDELKKAFAVIDQDKSGFIE 60
Cypc1.02    MAFAGVLNDADITAALEACKAADSFNHKTFFAKVGLTSKSADDVKKAFAIIDQDKSGFIE 60
PRVB        MAFAGLLTDADIAAAIDACSAADSFSHKTFFAKVGLSAKSADDVKKAFAIIDQDKSGFIE 60
PRVA1       MALAGLLAAADIDAALKACEAADSFKHKDFFAKVGLAAKSGDDVKKAFAIIDQDKSGFIE 60
            **:. :*  .** **:.**.***** ** **.**** .** *::*****:**********

PRVA        EDELKLFLQVFKKDARVLTDGETKAFLKAGDTDGDGKIGVDEFVAMVKA      109
Cypc1.02    EDELKLFLQNFKAGARALTDGETKTFLKAGDSDGDGKIGVDEFTALVKA      109
PRVB        EDELKLFLQNFKSGARALTDGETKTFLAAGDSDGDGKIGVDEFAALVKA      109
PRVA1       EEELKLFLQNFKAGARALTDTETKAFLKAGDSDGDGKIGVDEFAALIKP      109
            *:******* ** .**.*** ***:** ***:***********.*::*
```

B

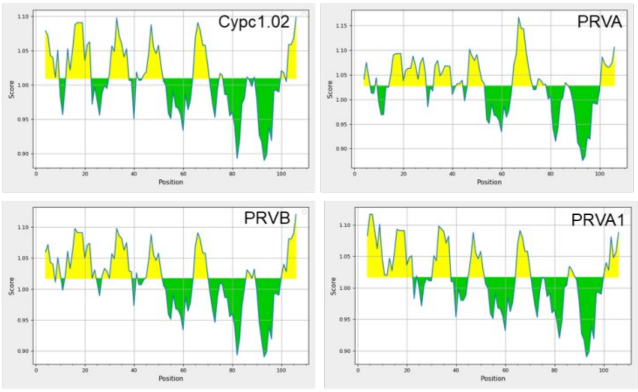


C

Cypc1.02

| Pos | Res | Start | End | Peptide | ES |
|---|---|---|---|---|---|
| 106 | L | 103 | 109 | FTALVKA | 1.099 |
| 14 | A | 11 | 17 | DITAALE | 1.022 |
| 16 | L | 13 | 19 | TAALEAC | 1.088 |
| 19 | C | 16 | 22 | LEACKAA | 1.091 |

PRVA

| Pos | Res | Start | End | Peptide | ES |
|---|---|---|---|---|---|
| 67 | F | 64 | 70 | LKLFLQV | 1.167 |
| 14 | A | 11 | 17 | DITAALD | 1.024 |
| 16 | L | 13 | 19 | TAALDAC | 1.09 |
| 19 | C | 16 | 22 | LDACKAA | 1.093 |

PRVB

| Pos | Res | Start | End | Peptide | ES |
|---|---|---|---|---|---|
| 106 | L | 103 | 109 | FAALVKA | 1.121 |
| 14 | A | 11 | 17 | DIAAAID | 1.033 |
| 16 | I | 13 | 19 | AAAIDAC | 1.098 |
| 19 | C | 16 | 22 | IDACSAA | 1.091 |

PRVA1

| Pos | Res | Start | End | Peptide | ES |
|---|---|---|---|---|---|
| 5 | G | 2 | 8 | ALAGLLA | 1.117 |
| 14 | A | 11 | 17 | DIDAALK | 1.027 |
| 16 | L | 13 | 19 | DAALKAC | 1.093 |
| 19 | C | 16 | 22 | LKACEAA | 1.091 |

*Pos: amino acid position; Res: Residue; ES: Epitope score*

**Fig S1.** In silico allergen potential of Hilsa PRVs.(A) The amino acid sequence alignment of Hilsa PRVs with well characterized common carp PRVB allergens Cycp 1.02. Red color amino acid in the sequence represents the potential epitopes. The red box shows the parvalbumin beta corresponding amino acids. B-C, the epitope region identified by IEDB (http://tools.iedb.org/bcell/) following Kolaskar and Tongaonkar method.
